# Chimeric antigen receptor-engineered T cells in CLL: the next chapter unfolds

**DOI:** 10.1186/s40425-016-0108-2

**Published:** 2016-02-16

**Authors:** Michael Kalos

**Affiliations:** Lilly Research Laboratories, Eli Lilly and Company, 450 East 29th street, New York, NY 10016 USA

**Keywords:** Immunotherapy, Chimeric antigen receptor, T cell, Leukemia adoptive transfer

## Abstract

The long-standing promise for the application of engineered T lymphocytes to target and eradicate malignancy has begun to be realized recently, with remarkable clinical success reported by a number of groups using Chimeric Antigen Receptor –engineered T cells to target CD19-positive hematologic malignancies. In the September 2 issue of Science Translational Medicine, Porter et al. present the clinical data and correlative analyses for 14 CLL patients treated at the University of Pennsylvania under the pilot clinical trial recently completed at that institution. The initial reports from this trial, published in 2011 documented robust clinical activity in a small cohort of treated patients accompanied by logarithmic expansion, contraction, and long-term functional persistence of engineered T cells, along with cytokine release syndrome as a side-effect of the treatment. In this latest report, updated data are presented from the initial cohort of patients, as well as clinical and correlative data from the remainder of the treated cohort. The robust clinical activity observed in the initial cohort continued to be observed in a subset of the subsequently-treated patients, with molecular remissions documented in that subset; however, in the expanded cohort a subset of partial and non-responding patients was also identified. Collectively, the results from this exciting trial provide evidence to suggest that cellular immunotherapy using engineered T cells is a viable option for treating CLL, reveal a likely requirement for robust in-vivo activation and persistence of engineered cells to effect complete responses, and also highlight the need for a more complete mechanistic understanding of the immune- and tumor- specific processes that define and dictate the success of this powerful treatment modality.

## Background

T lymphocytes, re-directed to target tumors through molecular engineering and expression of recombinant tumor-specific Chimeric Antigen Receptors (CAR) have shown remarkable promise in clinical trials that target hematological malignancies. Over the past 4 years, a series of high-profile reports by different groups have been published, demonstrating potent clinical activity using this treatment paradigm, including eradication of disease in late-stage patients with a variety of CD19-positive leukemias [1–11]. Among these efforts, the initial publications of the group from the University of Pennsylvania were particularly notable for the robust and sustained clinical activity in late-stage treatment refractory and relapsed CLL and ALL patients with heavy disease burden, and also for the systematic and mechanistically-informing biomarker strategy that was applied in those studies and which identified potential correlates for the observed clinical activities [1–3]. The initial reports focused on a small cohort of patients with late-stage disease, specifically 3 CLL patients and 2 pediatric ALL patients, each of whom had a strong anti-tumor response. Notable observations in these initial reports were the more than 4 log expansion of engineered cells in vivo, followed by contraction and long-term functional persistence of engineered cells, the deep molecular remissions of disease, as well as the development of cytokine release syndrome in each of the patients. For each of the initial reports, the small cohort sizes precluded any meaningful assessment of the clinical response rates, or a robust understanding of correlates with efficacy.

Initial reports from the NCI and Memorial Sloan Kettering groups confirmed the broader applicability of this approach [5, 9] and also extended the applicability of CAR-based targeting CD19+ malignancies to the setting of allogeneic transplantation [6] or as a bridge toward allogeneic transplantation [10]. More recent reports from the NCI group have also demonstrated the ability to potently target ALL as well as to effectively target additional CD19+ malignancies [7, 8]. Notable correlative observations from each of these reports were the apparent requirement for robust in-vivo expansion of engineered T cells for clinical activity, the observation of cytokine release syndromes as a correlate to clinical response, and, in contrast with the reports from the UPenn group, a lack of consistent long-term persistence of infused cells.

A more mature data set for the UPenn ALL patient cohort published earlier this year which included 25 pediatric and 5 adult patients demonstrated very high clinical activity with a 90 % complete response rate and 78 % overall survival at 6 months, and a robust set of correlative data to support the clinical observations [11]. In the September 2 issue of Science Translational Medicine [12], Porter et al. describe the mature clinical data and correlative analyses for the cohort of 14 CLL patients treated at the University of Pennsylvania. These expanded data further highlight and substantiate the potential for CAR-engineered T cell-based therapy to mediate profound activity in a subset of treatment refractory CLL patients, and also provide further insights into correlates of response post-infusion. Perhaps disappointingly given that more than one-third of treated patients did not respond to therapy, product- and patient- specific attributes predictive of response were not reported in these studies and remain undefined for CTL019 therapy of patients with advanced CLL.

## Main text

The expanded data set presented in this report enabled initial estimates of overall response rates to CTL019 for CLL patients, as well as the opportunity to begin to understand patient and product correlates of response. Estimated median survival for all treated patients was 29 months with 71 % overall survival at 18 months (95 % confidence interval of 40.6-88.2 %). No patient or tumor characteristics were identified that predicted or correlated with response. Cytokine release syndrome severity was associated with elevated peak levels of engineered cells and statistically-significant elevations in IL6 and IL2RA. Three general patterns of clinical response were observed in the 14 patient cohort: 4 patients achieved a complete response (CR), an additional 4 patients achieved a partial response (PR), and 6 patients did not respond (NR) to CTL019 treatment. For each of the 4 CR patients, robust and greater than 4-log expansion of CTL019 patients was observed, followed by contraction and functional persistence of CTL019 cells. Two of the CR patients were from the original cohort, and both these patients remained MRD-negative over 4 years post-treatment, with CTL019 cells detectable by Q-PCR and ongoing B cell aplasia. In the additional 2 CR patients CTL019 cells were also detectable in the periphery for over 12 months, and each of these patients remained MRD negative through the reporting interval. Partial responders were characterized by a more complex CTL019 expansion pattern: for 2 of the PR patients, in vivo expansion was considerably less than the CR cohort (2-3 logs); however, 2 PR patients had considerable expansion and persistence of CTL019 cells but did not resolve disease, suggesting the existence of CLL resistance mechanisms dominant to CTL019 activity; the MRD analysis in these patients suggested incomplete activity of CTL019 cells in marrow compared to the periphery at early timepoints. Non responders were characterized by lack of expansion, and no persistence, underscoring the requirement for some degree of in vivo functionality (proliferation, persistence) for CTL019 efficacy. Disappointingly, obvious and relevant questions about potential differences in product-specific attributes that might correlate with in vivo functionality were addressed only superficially in this report, with one set of data suggesting a “polyfunctional” cytokine response phenotype in all products, but not segregating these data in terms of clinical response or in vivo expansion properties despite a broad distribution in the reported data.

## Conclusions

The most notable conclusions from this report, which represents the most comprehensive data set with regard to CAR-based therapy in CLL are that i. CTL019 therapy has the potential to mediate compete molecular remissions in CLL; ii. Complete and overall response rates in CLL are significantly lower than those in ALL patients, and iii. Robust in-vivo expansion and sustained persistence of CTL019 cells are both likely requirements for potent anti-CLL activity. A fundamental and key outstanding question is whether the variability in response to CTL019 treatment can be uniquely attributed to a patient or a product specific property. The more uniform response rates in ALL together with the recently published observation that relapse mechanisms in ALL but not CLL are associated with CD19-splice variant selection [13] indicate that at least part of the variability of response in CLL disease can be attributed to in vivo disease-intrinsic mechanisms, although the impact of manufacturing a product using lymphocytes exposed to CLL vs ALL cells cannot be excluded. Efforts to better understand how CLL (a “chronic”) vs ALL (an “acute”) disease shape a T cell suppressive environment in vivo and also shape the functional properties of manufactured CTL019 cells by epigenetic or otherwise imprinted mechanisms are likely to be key to unlocking the full future potential of CTL019 therapy in CLL, by pointing the way to combination strategies to more effectively target this disease.
